# Antibiotic Resistance Trends Among Enterobacteriaceae in Saudi Arabia: A Systematic Review

**DOI:** 10.7759/cureus.56614

**Published:** 2024-03-21

**Authors:** Albandari A Arafah

**Affiliations:** 1 Microbiology, Fakeeh College of Medical Sciences, Makkah, SAU

**Keywords:** saudi arabia, enterobacteriaceae, bacteria, antimicrobial resistance, antibiotics resistance

## Abstract

Antibiotic resistance is a global public health concern that poses a significant threat to the effective treatment of bacterial infections. *Enterobacteriaceae*, a family of gram-negative bacteria, are associated with a wide range of infections, including urinary tract infections, bloodstream infections, and respiratory tract infections. This systematic review aimed to examine the antibiotic resistance trend among *Enterobacteriaceae *in Saudi Arabia in the period between 2003 and 2023. Five databases (PubMed, Medline, Ovid, Scopus, and Cochrane) were searched using the keywords “Resistance AND Enterobacteriaceae AND Saudi Arabia” in the title and abstract. All papers assessing the prevalence of resistance among *Enterobacteriaceae *in Saudi Arabia were included in the systematic review. Out of 97 papers that were extracted through the database search, 22 articles were considered suitable for the systematic review. The articles included 17027 *Enterobacteriaceae *isolates, out of which 7592 isolates were identified as resistant bacteria. The studies included various resistant strains, such as *Escherichia coli* and *Klebsiella pneumoniae*, that were responsible for various clinical conditions, including urinary tract infections, blood infections, surgical site infections, and pneumonia. In addition, the review highlighted the dynamic nature of antibiotic resistance, with the identification of new resistant bacterial species and the emergence of resistance to newer antibiotic classes over the last decade. Continued surveillance, rational antibiotic use, and the development of alternative treatment options are crucial to address the evolving landscape of antibiotic resistance among *Enterobacteriaceae *bacteria in the country.

## Introduction and background

Antibiotic resistance often arises within a few years of introducing a new antibiotic [1]. It occurs due to various mechanisms, including enzymatic inactivation or modification of antibiotics, alteration in bacterial target sites, permeability barriers to the antibiotic influx, active efflux pumps that extrude antibiotics from bacterial cells, and combinations of mechanisms [2-4]. Over time, mutations that confer resistance tend to increase, and antibiotic use can amplify this rate of increase due to selection pressure. Additionally, other factors that contribute to the spread of antimicrobial resistance (AMR) include crowding, poor hygiene, overuse and misuse of antibiotics, and increased travel [3].

Bacteria that belong to the *Enterobacteriaceae *family, such as *Enterobacter spp., Klebsiella spp., Escherichia coli (E. coli), Proteus spp., Serratia marcescens, and Citrobacter spp.*, can be found in the intestinal flora and can cause nosocomial infections. There are several treatments available for Enterobacter infections. These include penicillins and cephalosporins. Carbapenems, beta-lactamase inhibitors, fluoroquinolones, aminoglycosides, and sulfamethoxazole/trimethoprim [5]. In recent years, novel β-lactam/β-lactamase inhibitor combinations have been approved for the management of resistant organisms such as *Enterobacteriaceae *[6-8].

*Enterobacteriaceae *is most known for being antibiotic-resistant due to extended-spectrum β-lactamases (ESBLs) production, which can break down third-generation cephalosporins and aztreonam [9]. ESBL organisms are treated with carbapenem, the last resort to treat multidrug-resistant gram-negative bacteria. With the increase in carbapenem use, carbapenem-resistant Enterobacteriaceae (CRE) has become a public health concern [10]. The emergence and widespread dissemination of novel ESBLs and carbapenemases have contributed to a significant rise in AMR among *Enterobacteriaceae *worldwide over the past two decades [3,9,11,12].

Among carbapenemases-producing Enterobacteriaceae (CPEs), the most common resistance mechanism is the production of Klebsiella pneumoniae carbapenemase (KPC) enzymes. These enzymes are most frequently found in isolates of *Klebsiella pneumoniae (K. pneumoniae)*. Many countries around the world have recorded outbreaks caused by KPC-producing *K. pneumoniae* [13]. In Saudi Arabia, studies about AMR are limited and retrieved from separate institutions [14,15].

By systematically reviewing the available literature, the review will provide a comprehensive overview of the prevalence and extent of antibiotic resistance among *Enterobacteriaceae *in Saudi Arabia. This information will help identify the magnitude of the problem and contribute to the understanding of the current AMR landscape in the country. Therefore, our study aimed to investigate the antibiotic resistance trend among *Enterobacteriaceae *in Saudi Arabia from 2003 to 2023.

## Review

Methodology

This systematic review complied with established criteria (Preferred Reporting Items for Systematic Reviews and Meta-Analyses, PRISMA) [16].

Search Strategy

The systematic review was conducted through a thorough literature search of PubMed, Medline, Ovid, Scopus, and Cochrane databases using the keywords in the abstract and title: Resistance AND Enterobacteriaceae AND Saudi Arabia. One researcher screened studies published from 2003 to 2023 examining the antibiotic resistance trend among *Enterobacteriaceae *in Saudi Arabia to select studies that matched the inclusion and exclusion criteria.

Then, key data points were retrieved from the final record of the included research.

Inclusion and Exclusion Criteria

All papers assessing the prevalence of resistance among *Enterobacteriaceae *in Saudi Arabia were included in the systematic review. We excluded published studies in languages other than English, narrative reviews, duplicated papers, studies published before 2003 or conducted on the timeframe for bacterial resistance before 2002, studies with insufficient data or findings, studies with irrelevant findings, studies that did not include clinical samples and studies for which full text was unavailable.

Screening and Data Extraction

A reference manager was used to check the output of the search technique for duplication. The author first screened the titles and abstracts of the relevant studies. Then, relevant full-text papers were examined and evaluated for inclusion criteria. The data was independently extracted in a Microsoft Excel (Microsoft® Corp., Redmond, WA) spreadsheet. The data included authors, year of publication, study design and period, objective, methodology, population characteristics, and results of resistance pattern (bacterial species, antibiotics classes, mechanism of resistance).

Strategy for Data Synthesis

A summary table was created using data from relevant studies to provide a qualitative interpretation of the findings and study components.

Risk of Bias Assessment

In this systematic review, the risk of bias assessment was conducted among non-randomized studies of interventions (NRSI). We used the ROBINs-1 tool to assess NRSIs [17]. The assessments were conducted and the outcome assessed was the resistance pattern during 2002-2021. The judgment options were low, moderate, serious, and critical, and the overall risk of bias was reached using signaling questions. Issues that occurred while conducting the assessments due to unspecified study designs were corrected by discussion among the authors, and the most suitable judgment was agreed upon for these studies. The risk of bias revealed the overall quality of the included studies. Most studies need more reporting of study design details, including sample type, microbiological investigations, and antimicrobial susceptibility testing methods. Given the serious risk of bias observed within six of our included studies, our findings suggest the need for further investigational studies with more carefully designed and rigorously conducted studies involving larger sample sizes in the future.

Results

**Figure 1:**
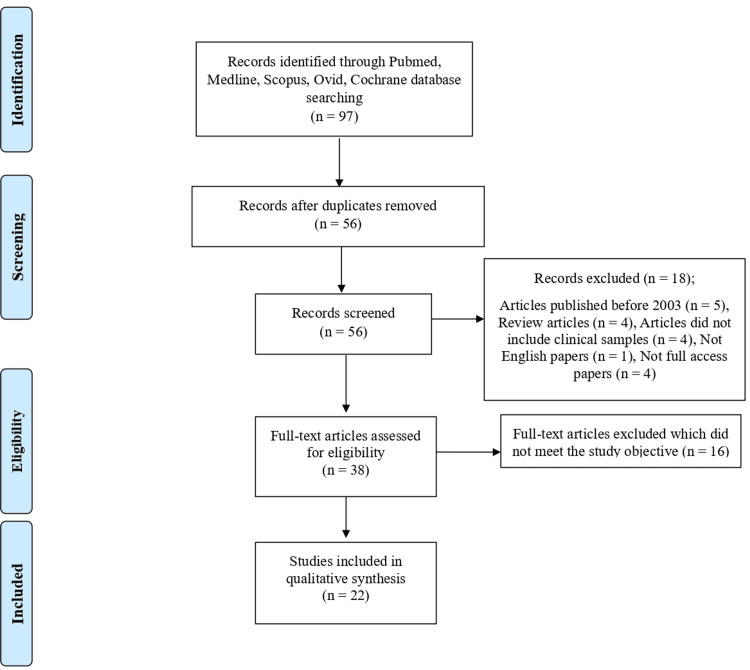
PRISMA flow diagram of study selection for the systematic review PRISMA: Preferred Reporting Items for Systematic Reviews and Meta-Analyses

As described in Figure 1, 22 articles were considered suitable for the systematic review.

Overview of the Included Studies

The included papers were published between 2004 and 2023 in different hospital settings in Saudi Arabia in different cities (Riyadh, Alkharj, Taif, Dammam, Jeddah, Alhassa, Bisha, Jazan, and Makkah) or regions (Western Region, Eastern Saudi Arabia, and Northern borders). The study duration was between 2002 and 2021 (Table 1). The study design varied among the included studies; four of the papers utilized a cross-sectional study design, three relied on retrospective analysis, two were prospective studies, and only one study involved a pooled analysis. Other studies did not report a definitive study design. The studies included diverse patient populations within the age range of 1 to 93 years, and most of them were intensive care patients.

**Table 1:** Characteristics of the included studies AMEs: aminoglycoside-modifying enzymes, *C. freundii: Citrobacter freundii*; CPKP: carbapenemase-producing Klebsiella pneumoniae; CRE: Carbapenem-resistant Enterobacteriaceae; CRPAE: carbapenem-resistant Pseudomonas aeruginosa; CSF: Cerebrospinal fluid; *E.coli: Escherichia coli*; ESBL: Extended Spectrum β-Lactamase; ER: Emergency room; ICU: Intensive care unit; *K. pneumoniae: Klebsiella pneumoniae*; MBL: Metallo-β-Lactamase; MDR: Multi-drug resistance; NA: Not available; *Proteus mirabilis: P. mirabilis*; PCR: polymerase chain reaction; OPD: Outpatient Department; pAmpc: plasmid-encoded extended spectrum β-lactamases; PMQR: Plasmid-Mediated Quinolone Resistance; UTIs: Urinary tract infections

Authors, year, location	Study design (Period)	Study objective	Methodology	Population characteristic	Results of Resistance pattern		
					Bacterial species	Antibiotic class	Mechanism of resistance
Kader and Kumar, 2004 [18]	NA (from March 2002 to June 2003)	To identify ESBL prevalence among MDR Enterobacteriaceae and non-fermenting gram-negative bacilli.	No. of collected specimens: 3231, Sources: Clean catch midstream or catheter urine, wound swabs, sputum and blood culture. Laboratory methods of resistance testing: Kirby-Bauer disc diffusion method	NA	Total no.: 156 Types: *E. coli* (72, 46%) *K. pneumoniae* (37, 21.6%), *Enterobacter sp.* (18, 11.5%), *Citrobacter sp.* (9, 5.8%)	More than 95% of the ESBL *E. coli* showed a minimum inhibitory concentration (MIC) of >256 mcg/ml against cefotaxime, ceftazidime and cefepime. In addition, 97% of the ESBL-producing *K. pneumoniae* had a high MIC value (>256 mcg/ml) against ceftazidime, cefotaxime, and cefepime.	Most bacteria were ESBL productive; the number of positive ESBL Enterobacteria was 136 (87%). *E. coli* (44%) and *K. pneumoniae* (24.6%)
Shibl et al., 2012 [19], Riyadh	NA (From July and December 2009)	To explore the prevalence of acquired quinolone resistance determinants among Enterobacteriaceae with ESBLs	No. of collected specimens: 600, Sources: NA Laboratory methods of resistance testing: Disc diffusion test or with E-tests.	NA	Total no. of bacteria with ESBLs: (160, 26.7%) Species: *E. coli* (99, 24.8%)* K. pneumoniae* (30.5%, 61)	*E-coli* isolates resistant to ciprofloxacin: 72.7% (72/99) *K. pneumoniae* isolates resistant to ciprofloxacin: 73.8% (45/61)	ESBL production
Marie et al., 2013 [20], Riyadh	NA, (July 2011 to October 2012)	To assess the prevalence of several β-lactamases characterized by PCRs.	No. of collected specimens: 4250, Sources: Blood, wounds, urine, sputum, and other body fluids. Laboratory methods of resistance testing: VITEK-2	NA	*E. coli* (3358, 79%) and *K. pneumoniae* (892, 21%).	The isolates showed the highest resistance to ciprofloxacin, followed by tobramycin, ceftriaxone, gentamicin, and amikacin. Furthermore, they showed a high resistance to carbapenem antibiotics antibiotics such as meropenem and imipenem. However, they were susceptible to colistin and tigecycline. ESBL strains were resistant to imipenem (15.5%), meropenem (15.5%), ciprofloxacin (28%), amikacin (30%), tobramycin (33%), and gentamicin (44%).	-Both MBL and ESBL were present in 22% of bacteria. -ESBL was detected more frequently in *E. coli* isolates. - Carbapenemase was identified more frequently in *Klebsiella pneumoniae* isolates.
Hassan et al., 2013 [21], (Eastern Saudi Arabia)	NA	To examine the ESBL-producing Enterobacteriaceae prevalence in eastern Saudi Arabia and to identify the ESBLs produced by these isolates at the molecular level.	No. of collected specimens: 236, Sources: Wound, urine, blood, sputum, CSF. Laboratory methods of resistance testing: VITEK-2 system (bioMerieux) Clinical setting: Ward, ICU, OPD, and ER	Age: Range: 0 - >60 years Gender: Male: (129, 54.7%) Female: (107, 45.3%)	Prevalence of ESBL-producing isolates: 4.8% (253). Bacterial species:* E. coli, Klebsiella spp.,* and *Proteus spp.*	The resistance rates to ceftazidime, cefotaxime, ceftriaxone, and aztreonam among *E. coli *isolates were 97.8%, 100%, 98.6%, and 98.5%, respectively, and among *K. pneumoniae* isolates were 96.6%, 97.7%, 95.3%, and 97.7% respectively. The resistance rate to the fourth-generation cephalosporine, cefepime, was 95.7% among *E. coli* and 91.8% among *K. pneumoniae* isolates. All *E. coli* and *K. pneumoniae* isolates were resistant to both piperacillin and cefazolin. Regarding the beta-lactam/beta-lactamase inhibitor combinations, the proportion of isolates showing resistance to amoxicillin/clavulanate (68.5%) was significantly higher than that showing resistance to piperacillin/tazobactam (41.1%).	The ESBL production
Al Sheikh et al., 2014 [22], Riyadh	NA, (Between January 2011 and December 2011)	To assess mechanisms of resistance among ESBLs Enterobacteriaceae.	No. of collected specimens: 33, Sources: Urine, blood, wounds, sputum, and other body fluids. Laboratory methods of resistance testing: VITEK-60 system (bioMerieux, Marcy l'Etoile, France)	NA	Total no. of bacteria with ESBLs: 218 Species: *E. coli* (50, 22.9%), *K. pneumoniae* (92, 42.2%), *C. freundii* (44, 20.2%), *Enterobacter spp* (32, 14.7%).	Ciprofloxacin (70%), Tobramycin (68%), Gentamicin (58%), Aztreonam (57%), Amikacin (54%), Cotrimoxazole (54%).	ESBL production
El-Hazmi, 2015 [23], Riyadh	Retrospective study (December 2009 to December 2011)	To investigate and examine the bacteriology of diabetic foot infection and their resistance patterns to antibiotics	No. of collected specimens: 268, Sources: wound swabs, tissue (including bone samples) and pus specimens from diabetic foot infections Laboratory methods of resistance identification: Automated system (Microscan Walkawa, Siemens) and confirmed by the disk diffusion	Number: 268 Gender: Male: 72.4%, Females: 27.6% Age: mean: 59.6 years.	*E. coli* (24, 10.4%).	Ampicillin (94%) Amoxicillin–clavulanic acid (71.6%) Cephalothin (73.1%) Cefuroxime (61.2%) Ceftriaxone (41.8%) Cefotaxime (41.8%) Cefepime (29.9%) Pipercillin- tazobactam (13.4%) Imipenem (2.2%) Merpenem (1.5%) Gentamicin (26.9%)	-19.4% of Enterobacteriaceae species were ESBL producers. -The rate of ESBL production in *E. coli *and *Klebsiella Spp.* was 53.3% and 27.6%, respectively.
Qamar et al., 2015 [24], Alkharj	NA, (February to September 2014)	To explore the resistant pattern of ESBL producing Enterobacteriaceae clinical isolates.	No. of collected specimens:131, Sources: Urine, Pus Sputum, Blood Laboratory methods of resistance identification: Vitek 2 method	NA	Enterobacteriaceae (84, 42%) with ESBL. *E. coli *(36, 42.85%) *Klebsiella* (23, 27.38%) *Proteus* (12, 14.28%) *Citrobacter* (8, 9.52%)	-Ampicillin (100%) -ceftazidime, trimethoprim/sulfa and norfloxacin antibiotics were the least effective antibiotics.	ESBL production
Alzahrani et al., 2016 [25], Taif city	NA, (Between February and August 2015)	To examine the antibiotic susceptibility of *E. coli* and* K. pneumoniae*. To detect common ESBL genes of the Enterobacteriaceae	No. of collected specimens: 43, Sources: Urinary tract infections, suppurative wounds in the perineum, sepsis of postoperative wounds. Laboratory methods of resistance testing: VITEK 2 (bioMérieux, Durham, NC, USA)	NA	Total no.: 17 (39.5%): Species: *E. coli* (14) and *K. pneumoniae* (3).	Ampicillin (17) Amoxicillin/ clavulanic acid (3) Piperacillin/ Tazobactam (7) Cefoxitin (3) Ceftazidime (17) Cefepime (17) Imipenem (0) Meropenem (0) Amikacin (1) Gentamicin (5) Ciprofloxacin (13) Tigecycline (0) Nitrofurantoin (4) Trimethoprim/ Sulfamethoxazole (11)	17 of 43 bacterial strains harbored genes for ESBL.
Abdalhamid et al., 2016 [26], Eastern Saudi Arabia	NA (From February 2015 to May 2015)	To examine the prevalence of intestinal carriage of CRE and CRPAE among patients admitted to ICUs in Saudi Arabia.	No. of collected specimens: 200, Sources: Rectal swabs Laboratory methods of resistance testing: VITEK 2 automatic system	Number: 200 Age: Median age: 43.8 years Range: 1–84 years. Clinical setting: ICU No of Enterobacteriaceae strains ()	Total number: 1 Species: *K. pneumoniae*	Imipenem (1/9 strains, 11.1%); Meropenem (1/9 strains, 11.1%); Ertapenem (1/9 strains, 11.1%); Cefepime (8/9 strains, 88.9%); Cefotaxime (8/9 strains, 88.9%); Ceftazidime (7/9 strains, 77.9%); Gentamicin (2/9 strains, 22.2%); Ciprofloxacin (2/9 strains, 22.2%)	ESBL production
Somily et al., 2016 [27], Riyadh	NA, (Between January 2011 and November 2013)	To compare phenotypic and molecular approaches for the identification and characterization of CRE isolates.	No. of collected specimens: 14, Sources: Wound, sterile body fluid, urine, blood, and respiratory.	Gender: Male (10, 76.9%) Female (3, 23.1%) Age: Range 1–93 Mean: 49 Clinical setting: Internal medicine (5, 38.5%) Surgical (4, 30.8%) Oncology (2, 15.4%) ICUs (2, 15.4%).	*E. coli* (2, 15.4%)* K. pneumoniae* (8, 61.5%) *K. oxytoca* (1, 7.7%) *E. cloacae* (2, 15.4%).	Amikacin (5/15 strains, 35.3%) Gentamicin (11/15, 73.3%); Trimethoprim-sulfamethoxazole (15/ 15 strains, 100%), Ciprofloxacin (9/ 15 strains, 60%)	1.6% of isolates were carbapenem-resistant. MBL Production. Among them, 5 were positive for the blaNDM gene and 3 were positive for the blaVIM gene.
Abdalhamid et al., 2017 [28], Eastern Saudi Arabia	NA (January to December 2015)	Examine the prevalence of pAmpC and its coexistence with ESBLs, PMQR, and AMEs in *E. coli, K. pneumoniae*, and *P. mirabilis* isolates in Saudi hospitals.	No. of collected specimens: 3625, Sources: Wound, respiratory, blood, and urinary tract specimens Laboratory methods of resistance identification: VITEK 2 system (bioMerieux)	No.: 200 Gender: Female (112) and Males (88) Age: mean age: 49.9 years (Rang: 1 - 86 years old). Clinical setting: ICU (123) and non-ICU patients (77)	Total no.: 200 Species: *E. coli* (108), *K. Pneumoniae *(80), and *P. mirabilis* (12)	Trimethoprim-sulfamethoxazole 88% (176/200) Ciprofloxacin 80% (160/200) Cefotaxime 78% (156/200) Ceftazidime 78% (156/200) Aminoglycosides 53.5% (107/200) Gentamicin 42% (84/200) Amikacin 42% (84/200) Meropenem 19% (38/200) Imipenem 24% (48/200) Ertapenem 24% (48/200)	Production of pAmpC b-lactamases and CMY-2 was the most prevalent pAmpC b lactamase.
Khan et al., 2019 [29]	Cross-sectional study (From January 2017 to December 2017).	To examine the association between carbapenemase emergence and enterobacterial infection.	No. of collected specimens: 120, Sources: Pus, urine, sputum, endotracheal tube, vaginal swab, peritoneal fluid Laboratory methods of resistance testing: Vitek-2 system	No.: 120 Gender: Female (112) and Males (88) Age: mean age: 24.6 ± 49.2 years. Range: 0.66 – 91 years Clinical setting: -ICU (24, 20%) -Medical ward (56, 46.7%) -Surgical ward (23, 19.2%) -Pediatric ward (12, 10%), Antenatal ward (1, 0.8%), Obstetrics and Gynecology (3, 2.5%), Outpatient (1, 0.8%).	Total no.: 26 Species: *K. pneumonia* (21, 80.8%) *E. cloacae* (2, 7.7%), *E. coli* (2, 7.7%) *P. mirabilis *(1, 3.8%30)	Ceftazidime (26, 100%), cefotaxime (26, 100%), ceftriaxone (26, 100%), cefepime (26, 100%), gentamicin (65.3%), amikacin (42.3%), Colistin (Nil).	Out of 17 isolates of CRE triple-resistant genes, KPC/NDM-1/OXA-48. Out of 4 isolates carried double resistant genes (KPC/OXA-48) or (NDM-1/OXA-48).
Aldrazi et al., 2020 [30], Dammam	NA	To find the pre of ESBL infections in Dammam Medical Complex, Eastern Province, Saudi Arabia	Total no.: 352 Sources: Pus, urine, Blood, Respiratory, CSF. Methodology: VITEK® 2 system	Sex Female: 122 Male: 230 Age: Range: from 0 to more than 80 years. Clinical setting: Burns Unit; Female Medical Ward; Female Surgical Wards; ICU; Male Medical Ward; Male Surgical Wards.	*K. pneumoniae* (148, 42.1%)* E.coli* (176, 50%), *P. mirabilis* (7, 2%), *Morganella morganii *(13, 3.7%), *Enterobacter* (7, 2%), *C. freundii* (1; 0.3%).	Trimethoprim/ Sulfamethoxazole (33.9%), Tigecycline (82.2%), Aztreonam (4.6%)	Production ESBL
Balkhy et al., 2020 [31], Riyadh, Jeddah, Alhassa and Dammam	Surveillance prospective study (from 2008 to 2016)	To identify data in a multi-hospital system in Saudi Arabia compared to the US National Health Surveillance Network.	No. of collected specimens: 1141.	No.: 37 Gender: Female 420 (45.4%) and Males 506 (54.6%) Age: Mean 40.7±29.7 Clinical setting: ICU, Step down unit, Specialty care areas, Wards, Outpatient clinics	*Klebsiella* (198, 15.7%), *Enterobacter *(122, 9.7%) *E. coli* (99, 7.9%).	- 34.3% of Klebsiella were resistant to third/ Fourth-generation cephalosporins -4.8% of Enterobacteriaceae were CRE.	NA
Balkhy et al., 2020 [32]	Pooled analysis (Between 2007 and 2016)	To examine ten-year resistance trends among pathogens causing healthcare-associated infections in a tertiary care setting in Saudi Arabia.	No. of specimens: 1544 pathogens Sources: Bloodstream infection, ventilator-associated pneumonia, catheter-associated urinary tract infections, dialysis access-related bloodstream infections, and surgical site infection.	Age: Mean: 43.4 ± 27.0 years	*Klebsiella spp. *(258, 14.7%) *Enterobacter spp.* (160, 9.1%)* E. coli *(159, 9.1%) *Serratia spp.* (40, 2.3%)	Acinetobacter: Aminoglycosides (50%); B-lactam (68.7%); Cephalosporins (77.9%); Fluoroquinolones (66.3%). Klebsiella: Aminoglycosides (32.9%); B-lactam (36.6%); Carbapenems (13.9%); Cephalosporins (43.1%); Fluoroquinolones (28.8%). Enterobacter: Aminoglycosides (10.5%); B-lactam (32.5%); Carbapenems (1.6%); Cephalosporins (50%); Fluoroquinolones (6.1%). E. coli: Aminoglycosides (34.8%); B-lactam (35.6%); Carbapenems (4.4%); Cephalosporins (52.4%); Fluoroquinolones (43.9%).	-Cephalosporin resistance *klebsiella* (32.1%). -CRE klebsiella (8.4%). - CRE *E.coli* (2.8%). - MDR* E.coli* (22.8%). MDR *Serratia* (12.5%).
Badger-Emeka et al., 2021 [33]	-	To explore the antimicrobial susceptibility pattern and clonal relatedness of *Klebsiella pneumoniae* isolates collected for a period of three years through pulse field gel electrophoresis.	No. of collected specimens: 78, Laboratory methods of resistance identification: VITEK 2	NA	*K.pneumoniae* (78)	Amoxicillin (100%), Ampicillin/sulbactam (96.4%), Amoxicillin/clavulanic acid (91%), Cefoxitin (82.6%), ceftazidime (83.3%), Aztreonam (80%), Ertapenem (5.5%), imipenem (23.1%), meropenem (28.2%).	- 98% were ESBL-KP, - 69% were CRE strains. - 72.5% comprised the carriage of two MBLs (SIM and IMP).
Ibrahim et al., 2021, Bisha [34]	Cross-sectional study (Between September 2017 and August 2018)	To assess the antibiotic susceptibility patterns and distribution of the resistance genes blaTEM, blaCTX-M, blaSHV, and blaOXA ESBL in MDR Enterobacteriaceae and Acinetobacter baumannii.	No. of collected specimens: 274, Sources: Body fluids, including urine, stool, sputum, etc. - Swabs from wounds, eye, umbilical and vagina. Laboratory methods of resistance identification: Kirby-Bauer disk diffusion method.	NA	Total number: 124 *K. pneumoniae* (63.5% MDR),* P. mirabilis* (54.8% MDR), and *E. coli* (51.8% MDR)	- *K. pneumoniae* was resistant to cefuroxime (98%), aztreonam (87%), trimethoprim/sulfa (87%), and cefotaxime (83%). -* E. coli *was resistant to trimethoprim/sulfamethoxazole (92%), cefuroxime (87%), and ceftazidime (71%). - *P. mirabilis* was resistant to trimethoprim/sulfamethoxazole (100%), amoxicillin/clavulanate (88%), cefotaxime, cefuroxime (88%), cefepime (82%), ciprofloxacin (82%) and ofloxacin (77%).	Out of 42.7% of the MDR, Enterobacteriaceae exhibited ESBL production.
Brek et al., 2023 [35], Jazan	Cross-sectional study (Between March 2020 and April 2021).	To evaluate the CPKP prevalence in the Jazan region, Saudi Arabia	No. of collected samples: 86 Sources: Urine (29.1%), sputum (24.4.%), blood (18.6%), wound (15.1%), intravascular tip culture (4.7%), bedsore (2.3%), endotracheal aspirate (2.3%), high vaginal swab (1.16%), peritoneal fluid (1.16%) and endotracheal tube tip (1.16%). Laboratory method of resistance testing: VITEK-2 system (BioMerieux, France)	Total number: 86 Gender: Male: 59, 68.6% Female: 27, 31.4 Clinical setting: Most of the bacteria (59; 68.6%) were isolated from ICU patients.	CRKP isolates (100%)	Amoxicillin-clavulanate (98.8%), piperacillin-tazobactam (90.7%), ceftazidime (95.3%), cefepime (95.3%), ciprofloxacin (91.9%), trimethoprim-sulfamethoxazole (89.5%), amikacin (82.6%), and gentamicin (79.1%), imipenem (57%), and tigecycline (20.9%).	Out of 64 (74.4%) isolates were carbapenemase-producing isolates. The blaOXA-48 gene was the most common carbapenemase gene (65.1%). The blaNDM gene was identified in 9.3% of isolates.
El-Masry et al., 2023 [36], Northern borders	Cross-sectional study (Between January to June 2021)	Determine the prevalence of Enterobacteriaceae clinical samples. Screening the antibiotics profile against the most used antimicrobials. Calculating the prevalence of ESBL among isolated samples	No. of collected specimens: 138, Sources: Stool, urine, wound, blood, tracheal aspirate, catheter tip, sputum, tracheal aspirate, and vaginal swab Laboratory methods of resistance identification: Disc diffusion method and VITEK 2 system (bioMerieux	Number: 37 Gender: Female (17) and Males (20) Age: NA Clinical setting: ICU (11) and non-ICU (26)	Total no. with ESΒL +ve: 37 Types: *E. coli* (19) *Klebsiella* (10) *Proteus* (8)	Amoxycillin (132, 95.7%) Azithromycin (128, 92.8%) Clindamycin (110, 79.7%) Imipenem (105, 76.1%) Ciprofloxacin (98,71.0%) Levofloxacin (91, 65.9%) Gentamycin (62, 44.9%) Trimethoprim-sulfamethoxazole (46, 33.3%) Tetracycline (37, 26.8%) Fosfomycin (21, 15.2%)	
Eltahlawi et al., 2023 [37], Jeddah	Prospective chart review (Between October 2020 and December 2021)	To examine the sensitivity of the Rapidec Carba NP test and GeneXpert Carba-R assay in comparison to conventional manners for identifying carbapenemase-producing Enterobacteriaceae.	No. of collected specimens: 90, Sources: Urine, Wounds, Swabs, Respiratory, Blood Sample, Sterile Body Fluid and Tissue. Most resistant bacteria were extracted from UTIs, followed by wound swab specimens, then respiratory tract infections and bloodstream infections. Laboratory methods of resistance identification: VITEK 2 system	Number: 90 Gender: Male: 51 (56.7%) Female: 39 (43.3%) Age: Mean: 51.14 (±23.8) Range: 1–88 Clinical setting: ICU isolates: 52 (57.8%) Non-ICU isolates: 38 (42.2%)	Total number: 90 Types: *K. pneumoniae*: (71, 78.9%) *K. Oxytoca*: (2, 2.2%) *E. coli*: (13, 14.4%) *E. aerogenes*: (2, 2.2%) *S. marcescens*:1 (1.1%) *C. freundii*:1 (1.1%)	*K. pneumonia*: Ceftriaxone (99%),Ceftazidime (99%), Cefepime (99%), Amoxicillin- clavulanate (99%), Piperacillin- tazobactam (99%), Imipenem (100%), Meropenem (100%), Gentamycin (68%), Amikacin (48%), Ciprofloxacin (95%), Trimethoprim/ Sulfamethoxazole (85%), Nitrofurantoin (84%), Tigecycline (9%). *K. oxytoca*: Ceftriaxone (100%),Ceftazidime (100%), Cefepime (100%), Amoxicillin- clavulanate (100%), Piperacillin- tazobactam (100%), Imipenem (100%), Meropenem (100%), Gentamycin (100%), Amikacin (100%), Ciprofloxacin (100%), Trimethoprim/ Sulfamethoxazole (100%), Nitrofurantoin (0%), Tigecycline (0%). *E. coli*: Ceftriaxone (92%), Ceftazidime (92%), Cefepime (92%), Amoxicillin- clavulanate (100%), Piperacillin- tazobactam (100%), Imipenem (100%), Meropenem (100%), Gentamycin (69%), Amikacin (46%), Ciprofloxacin (69%), Trimethoprim/ Sulfamethoxazole (77%), Nitrofurantoin (40%), Tigecycline (23%).* E. aerogenes*: Ceftriaxone (50%), Ceftazidime (50%), Cefepime (50%), Amoxicillin- clavulanate (100%), Piperacillin- tazobactam (100%), Imipenem (100%), Meropenem (100%), Gentamycin (50%), Amikacin (50%), Ciprofloxacin (50%), Trimethoprim/ Sulfamethoxazole (100%), Nitrofurantoin (100%), Tigecycline (0%). *S. marcescens*: Ceftriaxone (0%), Ceftazidime (0%), Cefepime (0%), Amoxicillin- clavulanate (100%), Piperacillin- tazobactam (100%), Imipenem (100%), Meropenem (100%), Gentamycin (0%), Amikacin (0%), Ciprofloxacin (100%), Trimethoprim/ Sulfamethoxazole (100%), Tigecycline (100%). *C. freundii*: Ceftriaxone (0%), Ceftazidime (0%), Cefepime (0%), Gentamycin (0%), Amikacin (0%), Ciprofloxacin (100%), Trimethoprim/ Sulfamethoxazole (100%), Tigecycline (0%).	Carbapenemase gene: - blaOXA-48 was the most predominant 44.4%, followed by blaNDM 32.2%.
Obaid et al., 2023 [38], Makkah region	A retrospective cohort study (From January 2017 to December 2020)	To examine the antimicrobial-resistant pathogens causing catheter urinary tract infections in the ICU.	No. of collected specimens: 393, Sources: indwelling urinary catheters Laboratory methods of resistance identification: NA	Number: 164 Gender: Women (91, 55.5%) Men (73, 44.5%) Age: Mean 63.5 years Clinical setting: ICU	- *K. pneumoniae* (8.5%) - *E. coli* (13.5%)	Total no. of resistance: 64 (19.8%). Penicillin G (12.0%), Amoxicillin/Clavulanic acid (11.9%), Ampicillin/Sulbactam (3.3%), Piperacillin/Tazobactam (10.7%), Cephalothin (1.1%), Cefazolin (1.1%), Cefoxitin (4.8%), Cefuroxime (2.3%), Cefotaxime (2.1%), Ceftazidime (4.1%), Ceftriaxone (0.7%), Cefepime (7.9%). Aztreonam (1.3%), Imipenem (6.9%), Meropenem (8.4%), Doripenem (0.5%), Ertapenem (1.5%), Teicoplanin (0.3%), Fosfomycin (1.1%), Colistin (2.1%), Tigecycline (0.5%), Clindamycin (0.3%), Erythromycin (0.3%), Tobramycin (6.1%), Gentamicin (13.2%), Amikacin (5.9%), Ciprofloxacin (16.5%), Levofloxacin (13.2%), Norfloxacin (6.9%), Trimethoprim/Sulfamethoxazole (16.1%) Nitrofurantoin (9.4%) Tetracycline (1.1%), Oxacillin (0.8%), and Vancomycin (1.0%)	-Production of ESBL. -Antimicrobial resistance was (62.0%.)
Taha et al., 2023 [39], Jeddah	Retrospective study (Between April 2017 and March 2019)	To examine the prevalence rate of CRE and to assess the types of carbapenemase genes.	No. of collected specimens: 180, Sources: Blood, Respiratory, Sputum, Swab, Urine, Wound, and Other.	Age: Mean (SD): 62.8 (18.6) Gender: Male (109, 60.6%) Females (71, 39.4%)	Total no.: 180 *K. pneumoniae* (167, 92.8%) *E. coli* (12, 6.7%) *Enterobacter* (1, 0.6%)	Piperacillin/Tazobactam (178, 98.9%) Meropenem (177, 98.3%) Ciprofloxacin (176, 97.8%) Imipenem (150, 83.3%) Amikacin (128, 71.1%) Tigecycline (37, 20.6%) Colistin (32, 17.8%)	-Carbapenemase-producing Enterobacteriaceae. The blaOXA-48 (76.1%) gene was prevalent among overall bacteria, followed by blaNDM (13.9%). Both genes coexisted in 6.1% of the isolates.

The articles included 17027 isolates of *Enterobacteriaceae *bacteria, out of which 7592 isolates were identified as resistant bacteria. The most frequently collected specimens were blood (15 studies), urine (13 studies), wound specimens (13 studies), sputum (10 studies), and pus (two studies). Additionally, other sources include vaginal swabs, cerebrospinal fluid, tissue, pus, respiratory infection, endotracheal tube, rectal swabs, and endotracheal tube. Some studies focused on specific patient groups, such as those with diabetic foot infections or catheter-associated urinary tract infections (UTIs).

Most studies depended on the VITEK-2 system (bioMérieux, Marcy-l'Etoile, France) in testing bacterial resistance. Among the *Enterobacteriaceae *bacteria, the most prevalent resistant species were *E. coli and K. pneumoniae*. Antibiotic resistance patterns varied among these bacteria, with resistance observed against multiple classes of antibiotics. The bacteria demonstrated resistance to several classes of penicillins (ampicillin, amoxicillin-clavulanic acid, and piperacillin/tazobactam), cephalosporins (cephalothin, ceftazidime, and cefepime), carbapenems (meropenem and imipenem/cilastatin), fluoroquinolones (ciprofloxacin), sulfonamides (trimethoprim-sulfamethoxazole), aminoglycosides (tobramycin and gentamicin), monobactam (aztreonam), and macrolides (azithromycin). However, tigecycline and colistin exhibited the lowest resistance rates among *Enterobacteriaceae *bacteria.

Moreover, ESBL production was a prominent focus in the included studies, with reported prevalence rates ranging from 1.6% to 87%. CREs were also identified and reported in several studies. Additionally, some studies highlighted the coexistence of multiple resistance mechanisms, such as the presence of both metallo-beta-lactamase (MBL) and ESBL in a subset of isolates. All details are described in Table 1.

Risk of Bias Assessment

The risk of bias revealed the overall quality of the included studies, according to the ROBINS-I tool (Table 2).

**Table 2:** Robvis Traffic Light Plot Figure Domains: D1: Bias due to confounding D2: Bias due to the selection of participants D3: Bias in the classification of interventions D4: Bias due to deviation from intended interventions D5: Bias due to missing data D6: Bias in measurements of outcomes D7: Bias in measurement of reported results Judgment + Low - Moderate x Serious Robvis: Risk-Of-Bias VISualization

Study ID	D1	D2	D3	D4	D5	D6	D7	Overall
Kader and Kumar, 2004 [18]	X	+	+	+	+	-	-	X
Shibl et al., 2012 [19]	X	X	+	-	+	+	+	X
Marie et al., 2013 [20]	X	+	+	+	+	+	+	X
Hassan et al., 2013 [21]	X	+	+	+	+	-	-	X
Al Sheikh et al., 2014 [22]	X	+	-	+	+	+	+	X
El-Hazmi, 2015 [23]	+	-	+	+	+	+	+	-
Qamar et al., 2015 [24]	-	-	+	+	+	+	+	-
Alzahrani et al., 2016 [25]	-	+	-	+	+	+	+	-
Abdalhamid et al., 2016 [26]	+	+	+	+	+	+	+	+
Somily et al., 2016 [27]	+	-	+	+	+	+	-	-
Abdalhamid et al., 2017 [28]	+	+	+	+	+	+	+	+
Khan et al., 2019 [29]	+	+	+	+	+	+	+	+
Aldrazi et al., 2020 [30]	+	+	+	+	+	+	+	+
Balkhy et al., 2020 [31]	+	+	+	+	-	-	-	-
Balkhy et al., 2020 [32]	-	+	+	+	-	-	-	-
Badger-Emeka et al., 2021 [33]	+	-	-	+	-	-	-	-
Ibrahim et al., 2021 [34]	+	-	+	+	X	-	-	X
Brek et al., 2023 [35]	+	+	+	+	+	+	+	+
El-Masry et al., 2023 [36]	+	-	+	+	-	+	+	-
Eltahlawi et al., 2023 [37]	+	+	+	+	+	+	+	+
Obaid et al., 2023 [38]	+	+	-	+	+	+	+	-
Taha et al., 2023 [39]	+	+	+	+	+	+	+	+

Discussion

AMR poses a serious public health emergency, which is primarily attributed to the overuse of antibiotics [40,41]. The resistance patterns observed in a specific strain of bacteria reflect a combination of inherent (intrinsic) and acquired resistance mechanisms. While intrinsic mechanisms are universally present, acquired mechanisms may exist only in certain geographical areas, leading to heterogeneous prevalence within those areas. Additionally, within a particular healthcare facility, only specific wards or units may be affected by these acquired mechanisms. Consequently, the implementation of effective surveillance, coupled with timely and accurate reporting of local epidemiology, plays a vital role in providing clinicians with crucial information for the appropriate management of patients [42]. Therefore, this systematic review aimed to investigate the antibiotic resistance trend among *Enterobacteriaceae *in Saudi Arabia from 2003 to 2023.

*Enterobacteriaceae *bacteria are widely distributed and have a broad range of hosts. These bacteria have the potential to cross-infect and transmit between medical staff and patients. Additionally, they can acquire genetic material, such as plasmids or transposons, from external sources, enabling the horizontal transfer of drug-resistant genes. This, in turn, contributes to the extensive dissemination of drug-resistant bacteria [43,44].

Bacteria that belong to the *Enterobacteriaceae *family are responsible for causing several nosocomial infections and community-acquired infections. Particularly, it contributes to UTIs, respiratory infections, osteomyelitis, soft tissue infections, and endocarditis [5]. We highlighted several species of *Enterobacteriaceae *that have been implicated in various clinical conditions in the last two decades. These infections included blood infections, catheter-associated UTIs and UTIs, diabetic foot infections, surgical site infections, and pneumonia.

*E. coli and K. pneumonia* are *Enterobacteriaceae*'s most frequently detected human pathogens. They cause infections, including cystitis, septicemia, pneumonia, pyelonephritis, meningitis, and peritonitis [45,46]. In the present review, the most detected bacterial strains among patients in the hospital setting were *E. coli and Klebsiella spp.* [18-39]. Other bacterial species were also isolated and identified, such as *Enterobacter* [18,22,30,32,36,39], *Proteus spp.* [21,24,28-30,36], *C. freundii* [22,30,37], *Citrobacter spp.* [24,30,36], *K. oxytoca *[27,37], *E. cloacae* [27,29], *Morganella morganii* [30,36], *Serratia spp.* [32,36,37], *P. mirabilis* [34], *Enterobacter aerogenes* [37]. The noteworthy observation is the identification of new resistant bacterial species over the last decade in the included studies (*Citrobacter spp.* [24,30,36], *K. oxytoca* [27,37], *E. cloacae* [27,29], *Morganella morganii* [30,36], *Serratia spp.* [32,36], *P. mirabilis* [34], *E. aerogenes* [37], and *S. marcescens* [37]). This suggests an evolving landscape of antibiotic resistance, emphasizing the dynamic nature of bacterial adaptation to antimicrobial agents.

It is a well-known fact that pathogens can develop drug resistance under the pressure of antibiotic selection. This can lead to a reduction in the effectiveness of antibiotics against infectious pathogens. Furthermore, the irrational use of antibiotics has resulted in many pathogens developing multidrug resistance, which is a cause of great concern in the medical community [47]. In recent years, the widespread utilization of β-lactamases has resulted in the elimination of the effectiveness of most cephalosporins against *Enterobacteriaceae*. As a result, carbapenems have become crucial in the treatment of clinical infections caused by these resistant bacteria. Unfortunately, the incidence of CREs has been steadily increasing over the years [48,49]. Among the included studies, ESBL production was frequently reported among *Enterobacteriaceae*, with prevalence rates ranging from 4.8% to 87% [18,20-24,26,30,33,34,38]. *Enterobacteriaceae *that harbored carbapenemase enzyme was detected in some studies [20,27,29,33,35,37,39]. On the other hand, the AmpC enzyme was identified among *Enterobacteriaceae *in only one study [28].

The traditional first-line antibiotics used to treat serious infections caused by *Enterobacteriaceae *are penicillins, cephalosporins, monobactams, carbapenems, fluoroquinolones, and, in specific cases, aminoglycosides [42]. Concerning our results, the bacteria demonstrated high resistance to several classes of penicillins (ampicillin, amoxicillin-clavulanic acid, and piperacillin/tazobactam), cephalosporins (cephalothin, ceftazidime, and cefepime), carbapenems (meropenem and imipenem), fluoroquinolones (ciprofloxacin), sulfonamides (trimethoprim-sulfamethoxazole), aminoglycosides (tobramycin and gentamicin), monobactam antibiotics (aztreonam), and macrolides (azithromycin).

Additionally, the trend of antibiotic resistance exhibited by bacterial strains varied across the years. It was detected that the bacterial strains were highly resistant to meropenem and imipenem [20], ciprofloxacin, tobramycin [22], ampicillin, amoxicillin-clavulanic acid, and cephalothin, cefuroxime [23] before 2013. On the other hand, in the last decade, the bacterial pathogens exhibited resistance to other antibiotics classes such as ceftazidime [24,25,29,33,35], trimethoprim/sulfa [24,25,33,35], norfloxacin [24], aminoglycosides [26,29,35], ceftriaxone [29], cefepime [29], tigecycline [30,35,37-39], aztreonam [33], ofloxacin [33], levofloxacin [35], piperacillin-tazobactam [35,39], amikacin [35], fosfomycin [35]. The shift in resistance patterns in the last decade, with bacterial strains showing resistance to newer antibiotic classes, indicates a dynamic adaptation of bacteria to the antibiotic landscape, which suggests a need for updated treatment guidelines and an awareness of emerging resistance to newer antimicrobial agents.

The trend of increasing the rate of resistance towards carbapenems over the years was noted in the included studies. In a study published in 2013, the resistance rate to both imipenem and meropenem was recorded at 15.5%. However, as time progressed, this resistance rate gradually climbed to a staggering 100% [20,33,35-39].

Despite colistin being considered the last-line drug for treating gram-negative bacteria [50], there has been a trend in resistance to colistin in recent years among the mentioned studies. Notably, a study conducted in 2017 revealed that all isolates were susceptible to colistin, indicating its effectiveness at that time [20]. However, subsequent studies conducted between 2017 and 2020 showed a significant rise in colistin resistance rates. One study reported a resistance rate of 2.1%, while another study found an alarming increase to 17.8% [38,39]. These findings highlight the growing challenge of colistin resistance and the urgent need for effective strategies to combat it.

One limitation of this study is that the included studies were limited to a specific time frame and geographical area (Saudi Arabia from 2003 to 2023). Therefore, the findings may not be representative of the global or long-term trends in antibiotic resistance among *Enterobacteriaceae*.

## Conclusions

The systematic review highlights the trend of antibiotic resistance among *Enterobacteriaceae *in Saudi Arabia. The studies included various resistant strains, such as *E. coli* and *K. pneumoniae*, that were responsible for various clinical conditions, including UTIs, blood infections, surgical site infections, and pneumonia. The review highlighted the dynamic nature of antibiotic resistance, with the identification of new resistant bacterial species and the emergence of resistance to newer antibiotic classes over the last decade. The study also revealed high resistance rates to several classes of antibiotics, including penicillins, cephalosporins, carbapenems, fluoroquinolones, sulfonamides, aminoglycosides, beta-lactam antibiotics, and macrolides. The emergence of new resistant bacterial species underscores the evolving landscape of antibiotic resistance and the need for continuous monitoring and adaptation of treatment guidelines. The findings emphasize the importance of rational antibiotic use and the development of alternative strategies to combat multidrug resistance.

Clinical implications

These findings highlight the urgent need for effective strategies to combat antibiotic resistance in Saudi Arabia. The data provide valuable insights into the prevailing resistance patterns and can guide healthcare professionals in selecting appropriate antimicrobial therapies. Continued surveillance, rational antibiotic use, and the development of alternative treatment options are crucial to address the evolving landscape of antibiotic resistance among *Enterobacteriaceae *bacteria in the country.
